# Cholesterol, high-density lipoprotein, and glucose (CHG) index and incident impaired fasting glucose in Chinese adults: a retrospective longitudinal cohort study

**DOI:** 10.3389/fnut.2026.1814747

**Published:** 2026-06-02

**Authors:** Duo Yang, Renzhe Lin, Sen Li, Shujun Ye, Zitian Luo, Huankai Zhang, Si Wu, Longsheng Zhang

**Affiliations:** 1 Department of Anesthesiology, Jieyang People’s Hospital, Jieyang, Guangdong Province, China; 2 First Clinical Medical College, Guangdong Medical University, Zhanjiang, Guangdong Province, China

**Keywords:** adults, cholesterol-high-density lipoprotein-and glucose-(CHG)-index, diabetes mellitus, impaired fasting glucose, insulin resistance

## Abstract

**Background:**

Diabetes mellitus poses a critical global public health burden, with impaired fasting glucose (IFG) being a crucial prediabetic state requiring early identification. The cholesterol, high-density lipoprotein, and glucose (CHG) index, a novel composite marker integrating TC, HDL-C, and FPG, has shown promise in assessing cardiometabolic risk. However, longitudinal evidence on its association with incident IFG, particularly in populations with normal baseline FPG, remains limited. This study aimed to evaluate the independent association between the baseline CHG index and the risk of incident IFG in Chinese adults.

**Methods:**

This retrospective longitudinal cohort study utilized data from 100,802 Chinese adults with normal baseline FPG, derived from a publicly available health screening cohort. The exposure was the baseline CHG index. The primary outcome was new-onset IFG. Multivariable Cox proportional hazards regression models were constructed to assess the association. Restricted cubic splines (RCS) were applied to explore potential nonlinearity, and subgroup analyses were conducted to evaluate the consistency of the association across different populations.

**Results:**

Over the follow-up period, 12,414 participants (12.32%) developed IFG. After full adjustment for covariates including demographic, anthropometric, clinical, and lifestyle factors, each one-unit increase in the CHG index was associated with a significantly increased risk of incident IFG. A clear dose–response relationship was observed across CHG index quartiles. RCS analysis revealed a significant nonlinear association with an inflection point at a CHG index of 5.259. Below this threshold, the risk increased sharply, while the association was attenuated and non-significant above it. This positive association remained consistent across all predefined subgroups.

**Conclusion:**

A higher baseline CHG index is an independent and strong predictor of incident IFG in Chinese adults with normal fasting glucose, exhibiting a nonlinear threshold relationship. The CHG index, easily calculated from routine lipid and glucose tests, may serve as a practical and economical tool for the early identification of individuals at high risk for prediabetes, informing targeted prevention strategies.

## Background

Diabetes mellitus (DM) has emerged as one of the most critical public health challenges worldwide in the 21st century. Its epidemiological characteristics are marked by a rapid increase in prevalence and a wide-reaching impact, imposing a heavy burden on healthcare systems. According to the latest data from the International Diabetes Federation (IDF), approximately 537 million adults globally are living with DM, and the prevalence continues to rise. It is projected that the total number of cases will increase to 783 million by 2045 and approach 900 million by 2050 (1, 2). China has the world’s largest population of individuals with DM, exceeding 118 million people, accounting for about 22% of the global diabetic population (3, 4). The country has the highest number of DM cases worldwide, alongside a substantial number of undiagnosed individuals, making the prevention and control situation extremely severe. The series of microvascular and macrovascular complications caused by DM, such as retinopathy, nephropathy, neuropathy, and cardiovascular and cerebrovascular diseases, not only severely impair patients’ quality of life but are also significant causes of disability and mortality, resulting in a substantial socioeconomic burden (5). Impaired fasting glucose (IFG) is a crucial state within the spectrum of prediabetes, regarded as an essential transitional phase from normal glucose homeostasis to DM. It is diagnosed when fasting plasma glucose (FPG) levels are elevated to a specific range but have not yet reached the diagnostic threshold for DM (6). IFG is not only a key risk factor for the progression from normal fasting glucose to overt DM, but the metabolic disturbances at this stage often already begin to cause harm to the body. For example, prolonged abnormalities in glucose metabolism are closely associated with an increased risk of both microvascular and macrovascular complications, such as cardiovascular disease (CVD), diabetic retinopathy (DR), and diabetic nephropathy (DN) (7, 8). Therefore, timely identification and intervention in high-risk individuals for IFG, while glucose metabolism is still in a potentially reversible early stage, are crucial for halting the progression to DM and reducing the incidence of cardiovascular events. Insulin resistance (IR) is widely recognized as the core pathophysiological basis for the development and progression of DM, and it serves as a key link connecting metabolic syndrome (MetS) with cardiovascular risk factors such as hypertension and dyslipidemia (9, 10). As insulin sensitivity decreases, the body’s ability to handle glucose is impaired, leading to compensatory hyperinsulinemia and elevated FPG levels. Although the hyperinsulinemic-euglycemic clamp technique remains the current gold standard for assessing IR, its complexity, high cost, and invasive nature make it difficult to implement routinely in low-income countries or for large-scale epidemiological screening (11). Consequently, there is an urgent clinical need to explore simple, economical, and readily obtainable alternative biomarkers from routine biochemical tests to accurately assess the degree of IR and predict the risk of early glucose metabolism abnormalities, such as IFG.

In recent years, researchers have been dedicated to developing simple surrogate indicators based on fasting blood biochemical tests. Among these, the triglyceride-glucose (TyG) index, serving as an effective surrogate marker for IR, has been widely adopted in the risk assessment of metabolic diseases, strongly supporting the pivotal role of glucolipotoxicity in pancreatic *β*-cell dysfunction (12, 13). However, dyslipidemia in the body typically manifests as complex alterations across multiple lipid species, and relying solely on triglyceride (TG) levels may fail to fully capture the complete pathophysiological picture underlying IR. Accumulating evidence suggests that, beyond TG, total cholesterol (TC) and high-density lipoprotein cholesterol (HDL-C) also play central roles in the regulation of glucose homeostasis (14). On one hand, hypercholesterolemia can directly lead to lipotoxic damage to pancreatic *β*-cells by inducing oxidative stress and mitochondrial dysfunction, thereby inhibiting insulin secretion (15). On the other hand, HDL-C is not only responsible for reverse cholesterol transport but is also crucial for maintaining normal insulin sensitivity through mechanisms such as anti-inflammatory and antioxidant properties, and promoting glucose uptake in skeletal muscle (16). Given the limitations of individual lipid components in reflecting intricate metabolic networks, constructing a composite index that integrates key metabolic parameters—FPG, TC, and HDL-C—could theoretically provide a more precise quantification of the disturbance in glucose-lipid metabolic coupling. This approach may offer superior predictive performance for early identification of high-risk individuals compared to traditional indicators (17, 18).

To overcome the limitations of single indicators and to more comprehensively capture the features of metabolic dysregulation, the cholesterol, high-density lipoprotein, and glucose (CHG) index has been developed by researchers. This index, derived from a specific formula, is designed to reflect the complex interplay between lipid metabolism and glucose homeostasis (19). As an emerging biomarker, the CHG index has garnered significant attention in the fields of cardiovascular and metabolic diseases in recent years. Current epidemiological evidence primarily focuses on the association between the CHG index and cardiovascular and cerebrovascular endpoints. Multiple large-scale cohort studies have demonstrated that an elevated baseline CHG index is an independent risk factor for an increased risk of CVD, ischemic stroke, and all-cause mortality. Notably, these associations remained significant even after adjusting for multiple confounding factors (18, 20). The CHG index has been confirmed to be strongly correlated with MetS and its components and is considered an effective surrogate marker for identifying IR and metabolic dysfunction (21). Specifically, in research on DM, several large-population cross-sectional analyses have shown that the CHG index exhibits excellent discriminative ability in diagnosing prevalent DM. Its diagnostic performance in some studies was even superior to or equivalent to that of the traditional TyG index (19). These findings suggest that the CHG index holds significant clinical value in identifying established severe metabolic diseases and predicting long-term adverse outcomes. Although the value of the CHG index in predicting CVD, stroke, and all-cause mortality has been confirmed in multiple cohort studies, and its ability to predict DM has been validated by several cross-sectional studies (22, 23), longitudinal evidence regarding the relationship between the CHG index and incident IFG remains limited. It is still unclear whether the CHG index can identify early-stage glucose metabolism abnormalities, and this requires further investigation for confirmation.

Existing studies on the relationship between the CHG index and glucose metabolism abnormalities have the following main limitations. First, most studies employed a cross-sectional design, which can only assess the correlation between a single measurement of CHG index and the prevalence of DM, failing to establish a causal temporal sequence (22, 23). Second, while some studies primarily focused on cardiovascular endpoints or the risk of complications (such as retinopathy and nephropathy) in patients with established DM (24, 25), there is a lack of long-term follow-up data specifically targeting individuals with normal baseline fasting glucose. Given that IFG represents the critical line of defense before the onset of DM and that lifestyle interventions at this stage offer the highest cost-effectiveness, elucidating whether an elevated CHG index can independently predict the pathological transition from normal fasting glucose to IFG holds significant clinical importance for addressing the current evidence gap in the primary prevention of metabolic diseases. Based on this background, the present study aims to systematically evaluate the independent association between the baseline CHG index and the risk of incident IFG in a Chinese adult population by conducting a retrospective longitudinal cohort study utilizing publicly available data from the Dryad database. This study primarily focuses on individuals with normal baseline glucose levels to investigate whether the CHG index can serve as an early biomarker for identifying individuals at high risk of transitioning to IFG before their FPG reaches the diagnostic threshold. Furthermore, through this large-scale cohort, we intend to validate the CHG index as a simple, economical, and readily accessible clinical tool, assessing its potential application value for the screening and risk stratification of prediabetes in the Chinese population.

## Methods

### Data source

The data for this study were sourced from the internationally recognized open science data platform, Dryad. This platform adheres to open-access principles and integrates publicly available scientific research data resources across multiple disciplines. The dataset utilized in this study is associated with a large-scale Chinese population cohort study published in *BMJ Open* in 2018, entitled “Association of body mass index and age with incident DM in Chinese adults: a population-based cohort study” (26). Researchers can access the original data via the Digital Object Identifier linked to this publication. This dataset encompasses longitudinal clinical follow-up records from participants in a health screening program between 2010 and 2016. In accordance with Dryad’s policies, the re-analysis of publicly available data to explore new scientific questions is permitted, provided it complies with ethical and legal frameworks. As the original study had already obtained ethical review approval from the Rich Healthcare Group Review Board in China, and this secondary analysis does not involve new subject intervention, re-application for ethical review or re-obtaining informed consent was not required. All procedures in this study strictly followed the ethical principles outlined in the Declaration of Helsinki and were fully compliant with relevant national laws, regulations, and institutional management guidelines.

### Study population

**Figure 1:**
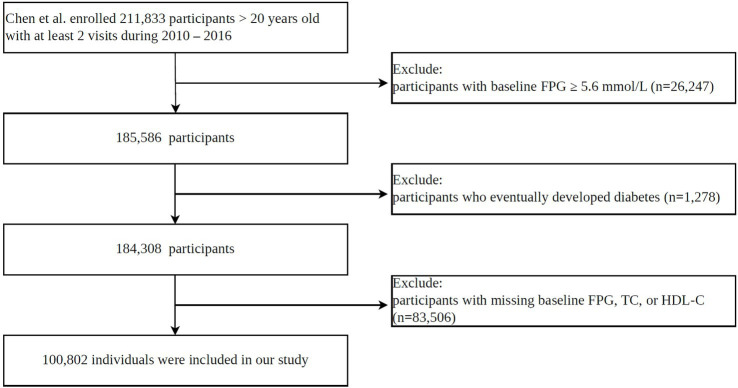
The detailed flowchart of this study.

This study constitutes a secondary analysis based on a cohort from mainland China. This cohort was originally established by Chen et al. and initially included 685,277 individuals. Based on predefined inclusion and exclusion criteria, a preliminary selection yielded 211,833 subjects, who served as the initial population for the present analysis (26). This study aimed to investigate the relationship between the baseline CHG index and the risk of incident IFG among individuals with normal baseline fasting glucose. To this end, further screening was applied to the initial population. First, individuals with a baseline FPG level ≥ 5.6 mmol/L were excluded. Second, cases diagnosed with DM during the follow-up period were removed. Finally, participants with missing data for key indicators such as TC, HDL-C, and FPG were excluded. Following these sequential steps, a total of 100,802 participants were ultimately included in the analysis phase. The detailed flowchart of the subject selection process is presented in Figure 1.

### Definitions of the exposure variable

The primary exposure in this study was the CHG index, treated as a continuous variable. The CHG index was calculated using the specific formula originally developed and validated by Mansoori et al. (19): CHG index = Ln [(TC × FPG) / (2 × HDL-C)]. Notably, the original formula requires the lipid and glucose parameters to be in mg/dL. Because our raw data for TC, FPG, and HDL-C were initially measured in mmol/L, we first converted these values to mg/dL (by multiplying FPG by 18, and both TC and HDL-C by 38.67) before applying the formula. This procedure ensures the exact reproducibility of the index as originally defined. This formula has been validated in studies of the Chinese population (18).

### Definitions of the outcome variable

The primary outcome of this study was defined as incident IFG. According to the relevant guidelines from the American Diabetes Association (ADA), IFG was diagnosed when a measured FPG level was ≥5.6 mmol/L and <7.0 mmol/L in an individual without a prior history of DM. Follow-up time was calculated from the date of the baseline health assessment to the date of the last available test for analysis.

### Other covariates

In this study, the following categories of covariates were included: (1) demographic characteristics, including age and gender; (2) anthropometric measurements, height, body weight, and blood pressure (BP); (3) blood biochemical parameters, covering FPG, TC, TG, low-density lipoprotein cholesterol (LDL-C), HDL-C, alanine aminotransferase (ALT), aspartate aminotransferase (AST), blood urea nitrogen (BUN), and serum creatinine (Scr); (4) behavioral and lifestyle factors, including smoking and drinking status; and (5) family history of DM.

Demographic information, behavioral/lifestyle factors, and family disease history were collected using structured questionnaires. Anthropometric measurements such as height, body weight, and BP were taken by uniformly trained staff following standardized operating procedures. Body mass index (BMI) was calculated as weight in kilograms divided by height in meters squared. Smoking and drinking status were categorized as “current,” “ever,” or “never” based on the classification criteria of the original database. Venous blood samples were drawn from all participants after a minimum fasting period of 10 h by professional personnel. All relevant biochemical parameters were measured using the Beckman Coulter AU5800 fully automated biochemical analysis system.

### Missing data processing

The dataset included in this analysis contained missing values for some variables. We addressed this issue following conventional strategies for observational epidemiological studies. Among the variables involved in the analysis, a total of 10 variables had missing records, comprising 8 continuous variables and 2 categorical variables. The specific proportions of missing data are detailed in Supplementary Table 1. Specifically, the missing percentages for the continuous variables were as follows: AST (58.04%), BUN (2.29%), Scr (1.21%), ALT (0.37%), LDL-C (0.16%), SBP (0.01%), DBP (0.01%), and TG (0.002%). To minimize potential bias introduced by missing data, we performed multiple imputation for the following continuous variables: ALT, AST, BUN, Scr, SBP, DBP, LDL-C, and TG. The procedure was implemented in the R statistical environment using the MICE package (27). The fundamental assumption behind the MICE method is that the data are missing at random (MAR), meaning that the probability of a value being missing depends on the observed data rather than the unobserved missing values themselves. Under this assumption, this method employed multiple imputation by chained equations with 5 iterations, generating 5 complete datasets.

For missing information in the categorical variables of smoking (72.43%) and drinking status (72.43%), these were uniformly assigned to a “Not recorded” category. To examine the reliability of the multiple imputation and verify the robustness of the study findings, the primary regression analyses reported in the main text were based on the pooled datasets after imputation. Furthermore, to comprehensively assess the stability of the conclusions, the results of analyses conducted solely on the original complete data (i.e., before imputation) and on data after excluding individuals with missing covariates are presented concurrently in the Supplementary material. The direction and magnitude of the association between the CHG index and the risk of incident IFG were essentially consistent across all three data handling approaches (after imputation, before imputation, and deletion of cases with missing covariates). This indicates that the main conclusions of this study are not sensitive to the choice of missing data handling method, demonstrating good stability of the results.

### Statistical method

**Figure 2:**
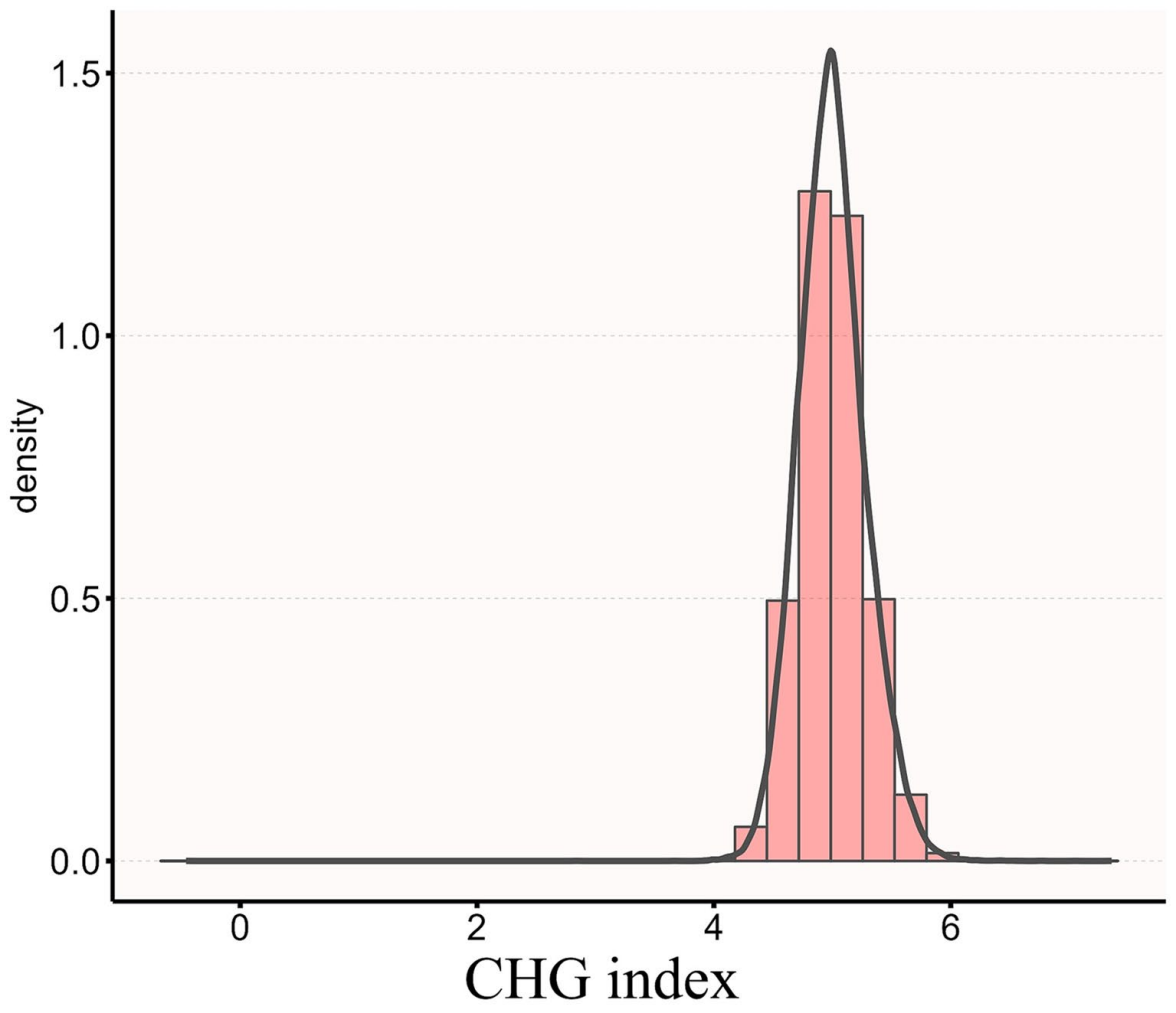
The distribution of the CHG index.

The distribution of the CHG index in the study population is presented in Figure 2. Based on the quartiles of the CHG index, all participants were divided into four groups: Q1 (≤4.811), Q2 (≤4.99), Q3 (≤5.174), and Q4 (≤7.358). The normality of continuous variables was assessed using the Kolmogorov–Smirnov test. Variables with a normal distribution are presented as mean ± standard deviation, while non-normally distributed variables are described as median (interquartile range) [M (IQR)]. Categorical variables are presented as frequency (percentage) [*n* (%)]. For between-group comparisons, one-way analysis of variance was used for normally distributed continuous variables with homogeneity of variance; otherwise, the Kruskal–Wallis H test was employed. Comparisons between categorical variables were performed using the chi-square test.

**Figure 3:**
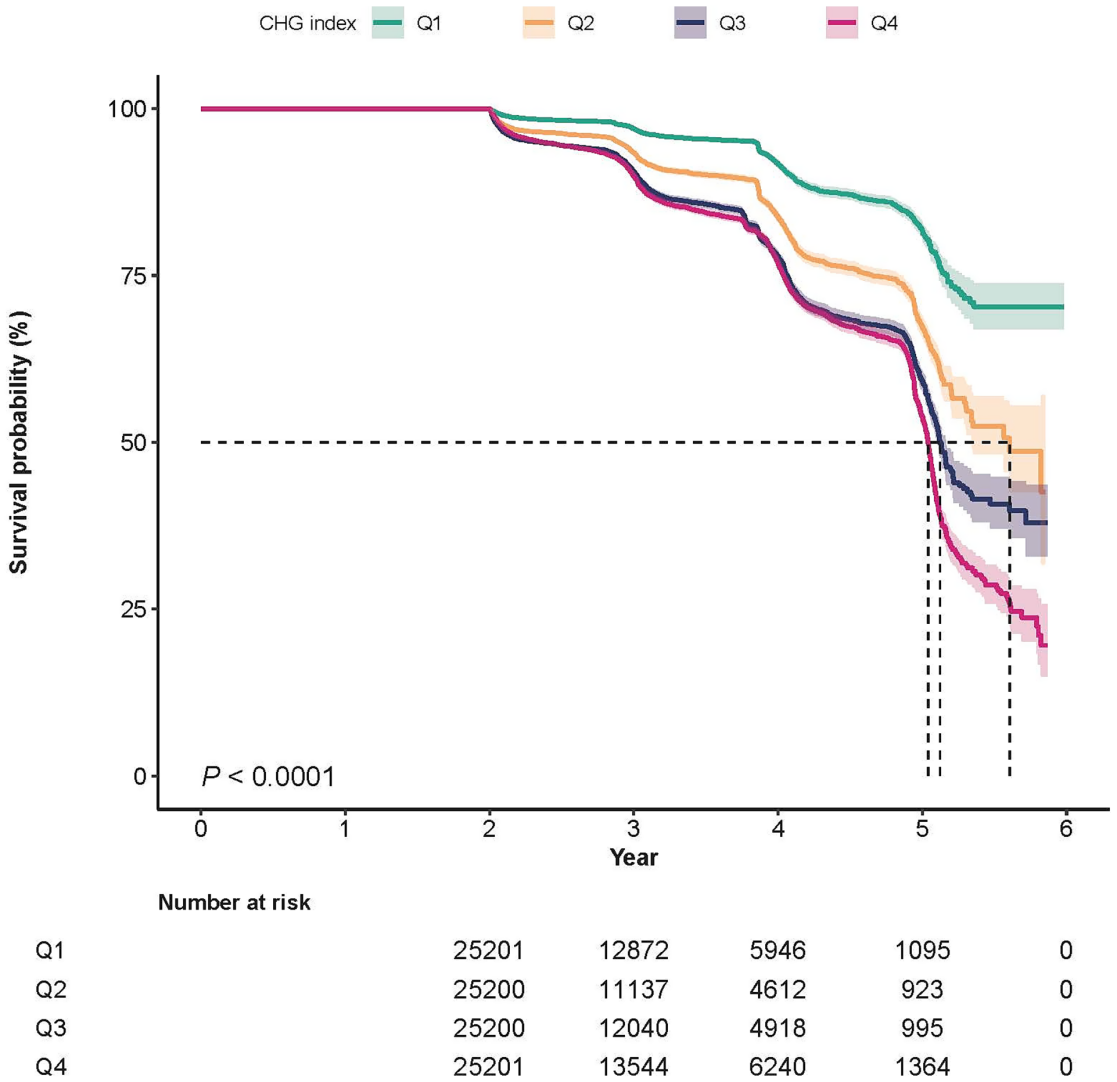
K-M curves illustrate incident IFG risk by CHG index quartiles. A pronounced divergence in survival curves was observed among the CHG index quartile groups (Q1 – Q4), with statistical significance confirmed by a log-rank test (*p* < 0.0001).

To explore the relationship between different levels of the CHG index and the risk of incident IFG, we plotted Kaplan–Meier survival function curves corresponding to the CHG index quartiles. The significance of differences between groups was assessed using the log-rank test. Furthermore, multivariable Cox proportional hazards regression models were constructed to evaluate the association between the CHG index and the risk of IFG, calculating hazard ratios (HRs) and their 95% confidence intervals (CIs). The proportional hazards (PH) assumption for the Cox models was evaluated (Supplementary Figure 1). Due to the exceptionally large sample size of our cohort (*N* > 100,000), the formal Schoenfeld residuals test yielded a *p*-value < 0.05, a well-documented phenomenon where even clinically negligible deviations reach statistical significance because of high statistical power. However, visual inspection of the Kaplan–Meier survival curves (Figure 3) revealed no crossing among the groups across the follow-up period, indicating no severe violation of the PH assumption. Consequently, the calculated hazard ratios in this study are interpreted as the time-averaged relative risks over the entire follow-up duration. To systematically examine the robustness of this association under varying levels of confounding factor control, we built two hierarchically adjusted regression models: Model I (partially adjusted model) was adjusted for age, gender, height, body weight, and family history of DM at baseline; Model II (fully adjusted model) was additionally adjusted for SBP, DBP, ALT, AST, TG, LDL-C, BUN, Scr, smoking status, and drinking status to fully control for potential confounding factors.

To enhance the robustness of causal inference and to examine the impact of different statistical models on the results, this study also concurrently constructed a multivariate logistic regression model to analyze the association between the CHG index and the prevalence of IFG. The adjustment variables included in this model were identical to those in the aforementioned Cox models. Consistent statistical conclusions from both the Cox proportional hazards regression model and the logistic regression model would significantly increase the credibility of the study findings.

Furthermore, to investigate the linear or nonlinear relationship between the CHG index and IFG risk in depth, restricted cubic splines (RCS) were applied to fit smooth curves. If the nonlinearity test indicated a significant nonlinear trend (*P* for nonlinearity < 0.05), a piecewise Cox proportional hazards regression model was further employed to identify a potential inflection point (threshold). The strength and direction of the association between the CHG index and IFG risk on either side of the inflection point were then estimated separately. Finally, subgroup analyses were conducted. The population was stratified by variables including gender, age, BMI, systolic blood pressure (SBP), diastolic blood pressure (DBP), LDL-C, HDL-C, TC, TG, smoking status, drinking status, and family history of DM. Cox regression analyses within each subgroup were uniformly adjusted for all other covariates except the stratification variable itself to assess the stability and consistency of the primary association across different population characteristics. To explicitly evaluate the predictive utility and overall performance of the models, we assessed both discrimination and calibration. Model discrimination was quantified using the Harrell’s Concordance Index (C-statistic). Furthermore, calibration plots were generated to visually assess the agreement between the model-predicted probabilities and the actual observed frequencies of incident IFG. Finally, the potential impact of unmeasured confounding factors was evaluated by calculating the E-value to quantitatively assess the robustness of the findings.

### Statistical software

All results are reported in accordance with the STROBE statement. Statistical analyses were performed in the R environment (version 4.2.2; https://www.R-project.org/) and the Free Statistics Analysis Platform (version 2.1.1; https://www.clinicalscientists.cn/freestatistics). A two-tailed *p*-value of less than 0.05 was considered statistically significant. As the present study constitutes a secondary analysis of pre-existing data, no formal *a priori* sample size calculation was performed.

## Results

### Baseline characteristics of the study population

This study included a total of 100,802 Chinese adults with normal baseline fasting glucose. The mean age was 42.90 ± 12.45 years, comprising 52,354 males (51.94%) and 48,448 females (48.06%). Participants were categorized into four groups (Q1 – Q4) based on quartiles of the baseline CHG index. As shown in Table 1, with increasing CHG index quartiles, there were significant increases in age, height, body weight, BMI, SBP, DBP, baseline FPG, TC, TG, LDL-C, ALT, AST, BUN, and Scr levels (all *p* < 0.001). Conversely, HDL-C levels showed a significant decrease (*p* < 0.001). Furthermore, the proportions of males, current smokers, and current drinkers were higher in the groups with higher CHG index values (all *p* < 0.001). However, the distribution of family history of DM did not differ significantly across the groups (*p* = 0.095).

**Table 1:** Baseline characteristics of the study population by CHG index quartiles.

Variables	Total (*n* = 100,802)	CHG index				
		Q1 (*n* = 25,201)	Q2 (*n* = 25,200)	Q3 (*n* = 25,200)	Q4 (*n* = 25,201)	*p*-value
CHG index, Mean ± SD	5.00 ± 0.28	4.66 ± 0.13	4.90 ± 0.05	5.08 ± 0.05	5.36 ± 0.16	< 0.001
Age (years), Mean ± SD	42.90 ± 12.45	38.98 ± 10.74	41.71 ± 12.02	44.13 ± 12.63	46.78 ± 12.94	< 0.001
Gender, *n* (%)						< 0.001
Male	52,354 (51.94)	8,531 (33.85)	11,790 (46.79)	14,483 (57.47)	17,550 (69.64)	
Female	48,448 (48.06)	16,670 (66.15)	13,410 (53.21)	10,717 (42.53)	7,651 (30.36)	
Height (cm), Mean ± SD	166.23 ± 8.30	164.66 ± 7.83	165.63 ± 8.30	166.63 ± 8.38	168.02 ± 8.30	< 0.001
Body weight (kg), Mean ± SD	64.12 ± 11.91	58.27 ± 9.94	62.26 ± 11.01	65.92 ± 11.55	70.03 ± 11.76	< 0.001
BMI (kg/m^2^), Mean ± SD	23.09 ± 3.22	21.41 ± 2.74	22.60 ± 3.01	23.64 ± 3.10	24.71 ± 3.06	< 0.001
SBP (mmHg), Mean ± SD	118.05 ± 16.08	112.81 ± 14.49	116.74 ± 15.51	119.98 ± 16.24	122.68 ± 16.30	< 0.001
DBP (mmHg), Mean ± SD	73.74 ± 10.77	70.58 ± 9.97	72.74 ± 10.44	74.80 ± 10.77	76.86 ± 10.84	< 0.001
Baseline FPG (mmol/L), Mean ± SD	4.78 ± 0.48	4.47 ± 0.49	4.76 ± 0.43	4.90 ± 0.41	5.00 ± 0.39	< 0.001
TC (mmol/L), Mean ± SD	4.75 ± 0.88	4.25 ± 0.71	4.53 ± 0.72	4.84 ± 0.77	5.36 ± 0.91	< 0.001
TG (mmol/L), M (IQR)	1.06 (0.73, 1.58)	0.75 (0.58, 1.00)	0.92 (0.70, 1.27)	1.19 (0.85, 1.64)	1.65 (1.19, 2.32)	< 0.001
HDL-C (mmol/L), Mean ± SD	1.38 ± 0.30	1.62 ± 0.30	1.44 ± 0.25	1.33 ± 0.22	1.13 ± 0.22	< 0.001
LDL-C (mmol/L), Mean ± SD	2.74 ± 0.67	2.31 ± 0.49	2.59 ± 0.50	2.84 ± 0.56	3.22 ± 0.73	< 0.001
ALT (U/L), M (IQR)	17.70 (12.70, 26.50)	14.10 (11.00, 20.00)	16.00 (12.00, 23.60)	18.90 (13.40, 27.90)	23.00 (16.00, 34.50)	< 0.001
AST (U/L), M (IQR)	21.90 (18.30, 26.00)	20.40 (17.70, 24.00)	21.00 (18.00, 25.20)	22.00 (19.00, 26.90)	23.20 (19.90, 28.60)	< 0.001
BUN (mmol/L), Mean ± SD	4.63 ± 1.16	4.40 ± 1.11	4.61 ± 1.15	4.73 ± 1.18	4.79 ± 1.15	< 0.001
Scr (μmol/L), Mean ± SD	69.83 ± 15.72	64.56 ± 14.02	68.82 ± 15.92	72.13 ± 16.00	73.83 ± 15.24	< 0.001
Smoking status, *n* (%)						< 0.001
Current smoker	5,370 (5.33)	677 (2.69)	998 (3.96)	1,415 (5.62)	2,280 (9.05)	
Ever smoker	1,097 (1.09)	152 (0.6)	224 (0.89)	319 (1.27)	402 (1.6)	
Never smoker	21,320 (21.15)	5,402 (21.44)	5,209 (20.67)	5,229 (20.75)	5,480 (21.75)	
Not recorded	73,015 (72.43)	18,970 (75.27)	18,769 (74.48)	18,237 (72.37)	17,039 (67.61)	
Drinking status, *n* (%)						< 0.001
Current drinker	646 (0.64)	74 (0.29)	148 (0.59)	180 (0.71)	244 (0.97)	
Ever drinker	4,570 (4.53)	743 (2.95)	969 (3.85)	1,316 (5.22)	1,542 (6.12)	
Never drinker	22,571 (22.39)	5,414 (21.48)	5,314 (21.09)	5,467 (21.69)	6,376 (25.3)	
Not recorded	73,015 (72.43)	18,970 (75.27)	18,769 (74.48)	18,237 (72.37)	17,039 (67.61)	
Family history of DM, *n* (%)						0.095
No	98,579 (97.79)	24,651 (97.82)	24,652 (97.83)	24,678 (97.93)	24,598 (97.61)	
Yes	2,223 (2.21)	550 (2.18)	548 (2.17)	522 (2.07)	603 (2.39)	
IFG, *n* (%)						< 0.001
No	88,388 (87.68)	23,776 (94.35)	22,718 (90.15)	21,487 (85.27)	20,407 (80.98)	
Yes	12,414 (12.32)	1,425 (5.65)	2,482 (9.85)	3,713 (14.73)	4,794 (19.02)	

During the follow-up period, a total of 12,414 participants (12.32%) developed incident IFG. As presented in Table 2, compared to participants who did not develop IFG, those with incident IFG had a higher baseline CHG index (5.12 ± 0.26 *vs.* 4.98 ± 0.28, *p* < 0.001). They were also older, had a higher proportion of males, and exhibited greater height, body weight, BMI, SBP, DBP, baseline FPG, TC, TG, LDL-C, ALT, AST, BUN, and Scr levels, while HDL-C levels were lower (all *p* < 0.001). The incident IFG group also had higher proportions of former smokers, current drinkers, and individuals with a family history of DM.

**Table 2:** Baseline characteristics of the study population by IFG outcome.

Variables	Non-IFG (*n* = 88,388)	IFG (*n* = 12,414)	*p* value
CHG index, Mean ± SD	4.98 ± 0.28	5.12 ± 0.26	< 0.001
Age (years), Mean ± SD	42.05 ± 12.04	48.99 ± 13.59	< 0.001
Gender, *n* (%)			< 0.001
Male	44,581 (50.44)	7,773 (62.61)	
Female	43,807 (49.56)	4,641 (37.39)	
Height (cm), Mean ± SD	166.17 ± 8.28	166.69 ± 8.42	< 0.001
Body weight (kg), Mean ± SD	63.57 ± 11.81	68.02 ± 11.92	< 0.001
BMI (kg/m^2^), Mean ± SD	22.91 ± 3.17	24.38 ± 3.25	< 0.001
SBP (mmHg), Mean ± SD	117.06 ± 15.61	125.14 ± 17.51	< 0.001
DBP (mmHg), Mean ± SD	73.20 ± 10.57	77.59 ± 11.37	< 0.001
Baseline FPG (mmol/L), Mean ± SD	4.75 ± 0.48	5.03 ± 0.41	< 0.001
TC (mmol/L), Mean ± SD	4.72 ± 0.88	4.92 ± 0.90	< 0.001
TG (mmol/L), M (IQR)	1.02 (0.71, 1.52)	1.30 (0.90, 1.94)	< 0.001
HDL-C (mmol/L), Mean ± SD	1.39 ± 0.31	1.34 ± 0.29	< 0.001
LDL-C (mmol/L), Mean ± SD	2.73 ± 0.67	2.84 ± 0.67	< 0.001
ALT (U/L), M (IQR)	17.10 (12.40, 26.00)	21.00 (14.70, 31.00)	< 0.001
AST (U/L), M (IQR)	21.60 (18.10, 26.00)	23.00 (19.10, 28.00)	< 0.001
BUN (mmol/L), Mean ± SD	4.60 ± 1.16	4.84 ± 1.17	< 0.001
Scr (μmol/L), Mean ± SD	69.37 ± 15.67	73.10 ± 15.69	< 0.001
Smoking status, *n* (%)			< 0.001
Current smoker	4,526 (5.12)	844 (6.8)	
Ever smoker	941 (1.06)	156 (1.26)	
Never smoker	18,977 (21.47)	2,343 (18.87)	
Not recorded	63,944 (72.34)	9,071 (73.07)	
Drinking status, *n* (%)			< 0.001
Current drinker	545 (0.62)	101 (0.81)	
Ever drinker	3,963 (4.48)	607 (4.89)	
Never drinker	19,936 (22.56)	2,635 (21.23)	
Not recorded	63,944 (72.34)	9,071 (73.07)	
Family history of DM, *n* (%)			0.01
No	86,478 (97.84)	12,101 (97.48)	
Yes	1910 (2.16)	313 (2.52)	

**Figure 4:**
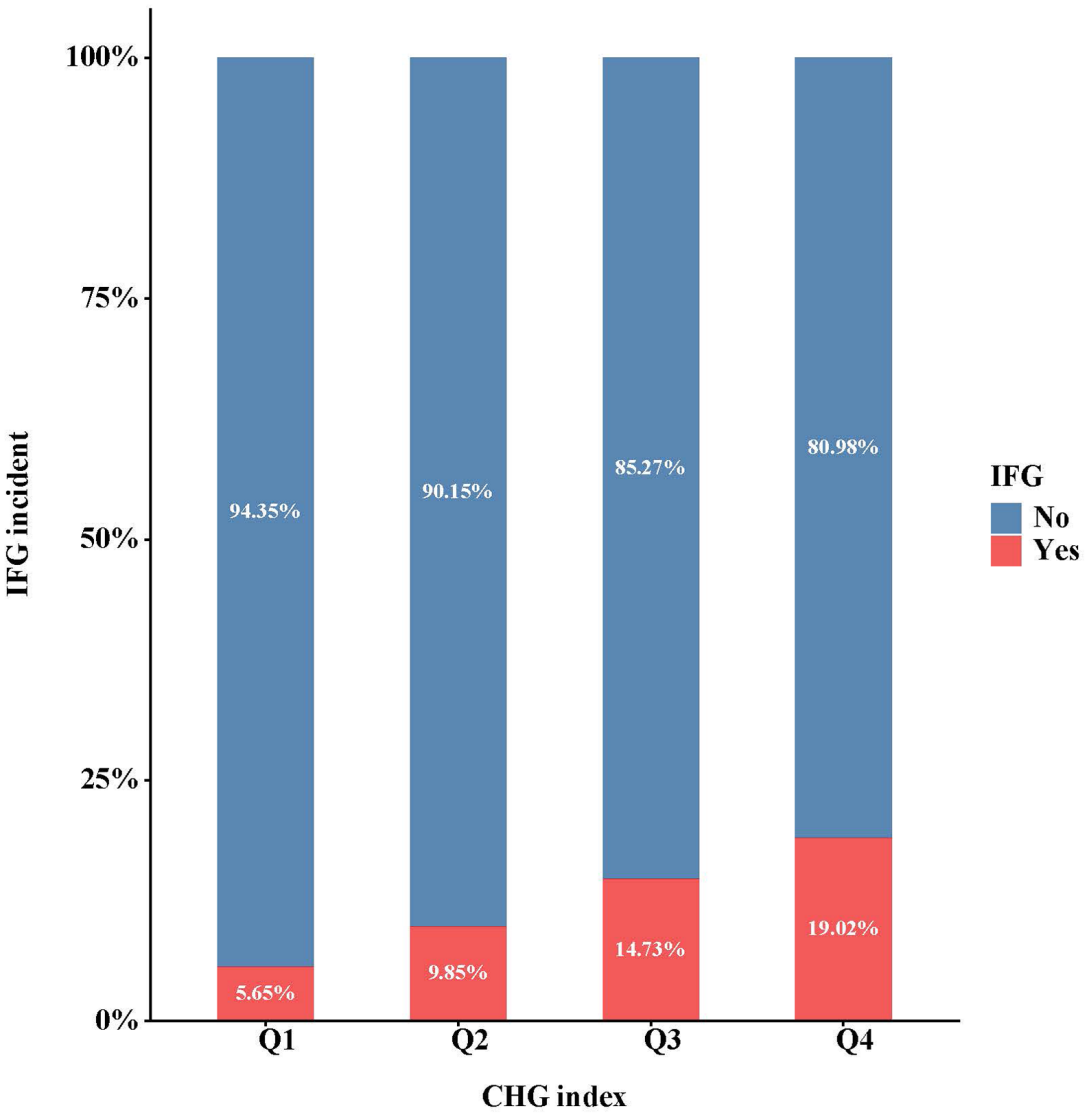
Stacked column chart of IFG incidence. The horizontal axis represents the CHG index quartile groups, and the vertical axis depicts the incidence rate of IFG (%). Blue indicates participants who did not develop IFG, and red indicates those who did develop IFG.

As shown in Figure 4, the stacked bar chart based on baseline CHG index groups visually displays the distribution of incident IFG across different CHG index levels. With ascending CHG index levels (from Q1 to Q4), the proportion of participants who developed IFG demonstrated a clear and graded increasing trend, with rates of 5.65%, 9.85%, 14.73%, and 19.02%, respectively. Correspondingly, the proportion of participants who maintained normal fasting glucose decreased progressively across these groups. A higher baseline CHG index was associated with a greater proportion of IFG development during follow-up.

### Association between the CHG index and the risk of incident IFG

Analysis using Cox proportional hazards regression models demonstrated that in the crude model without adjusting for any covariates, a higher CHG index was associated with a significantly increased risk of incident IFG (HR = 3.21, 95% CI: 3.04–3.40). This positive association remained significant after adjusting for age, gender, height, body weight, and family history of DM (Model I), and after further adjustment for blood pressure, liver function, lipid profiles, renal function, and smoking and drinking status (Model II), with HRs of 1.83 (95% CI: 1.71–1.95) and 1.93 (95% CI: 1.78–2.09), respectively (all *p* < 0.001) (Table 3).

**Table 3:** Association between CHG index and risk of incident IFG using Cox proportional hazards regression in different models.

Variables	Crude model		Model I		Model II	
	HR (95% CI)	*p* value	HR (95% CI)	*p* value	HR (95% CI)	*p* value
CHG index	3.21 (3.04, 3.4)	< 0.001	1.83 (1.71, 1.95)	< 0.001	1.93 (1.78, 2.09)	< 0.001
(CHG index quartiles)						
Q1	1.00 (Reference)		1.00 (Reference)		1.00 (Reference)	
Q2	2.05 (1.92, 2.19)	< 0.001	1.71 (1.6, 1.83)	< 0.001	1.7 (1.59, 1.82)	< 0.001
Q3	2.9 (2.73, 3.08)	< 0.001	2.11 (1.98, 2.25)	< 0.001	2.09 (1.96, 2.23)	< 0.001
Q4	3.17 (2.99, 3.37)	< 0.001	1.96 (1.84, 2.09)	< 0.001	1.96 (1.82, 2.1)	< 0.001
*P* for trend		< 0.001		< 0.001		< 0.001

Crude model: we did not adjust other covariates.

Model I: adjusted for age, gender, height, body weight, and family history of DM at baseline.

Model II: further adjusted for SBP, DBP, ALT, AST, TG, LDL-C, BUN, Scr, smoking status, and drinking status at baseline.

Analysis by CHG index quartiles showed that compared to the Q1 group, the risk of developing IFG gradually increased in the Q2, Q3, and Q4 groups, with a significant dose–response trend (all *P* for trend < 0.001). In the Model II, the HRs for the Q2, Q3, and Q4 groups were 1.70 (95% CI: 1.59–1.82), 2.09 (95% CI: 1.96–2.23), and 1.96 (95% CI: 1.82–2.10), respectively.

Logistic regression model analysis yielded results consistent with the Cox models. In the crude model, Model I, and Model II, for each one-unit increase in the CHG index, the odds ratios (ORs) for IFG risk were 5.26 (95% CI: 4.92–5.62), 2.60 (95% CI: 2.41–2.80), and 3.69 (95% CI: 3.36–4.06), respectively (all *p* < 0.001) (Table 4). Quartile analysis similarly revealed a significant graded increase in risk and a significant trend (all *P* for trend < 0.001).

**Table 4:** Association between CHG index and incident IFG, assessed using logistic regression in different models.

Variables	Crude model		Model I		Model II	
	OR (95% CI)	*p* value	OR (95% CI)	*p* value	OR (95% CI)	*p* value
CHG index	5.26 (4.92, 5.62)	< 0.001	2.6 (2.41, 2.8)	< 0.001	3.69 (3.36, 4.06)	< 0.001
(CHG index quartiles)						
Q1	1.00 (Reference)		1.00 (Reference)		1.00 (Reference)	
Q2	1.82 (1.7, 1.95)	< 0.001	1.47 (1.37, 1.58)	< 0.001	1.54 (1.44, 1.66)	< 0.001
Q3	2.88 (2.7, 3.07)	< 0.001	1.96 (1.83, 2.1)	< 0.001	2.14 (2, 2.3)	< 0.001
Q4	3.92 (3.68, 4.17)	< 0.001	2.22 (2.07, 2.37)	< 0.001	2.61 (2.42, 2.82)	< 0.001
*P* for trend		< 0.001		< 0.001		< 0.001

Crude model: we did not adjust other covariates.

Model I: adjusted for age, gender, height, body weight, and family history of DM at baseline.

Model II: further adjusted for SBP, DBP, ALT, AST, TG, LDL-C, BUN, Scr, smoking status, and drinking status at baseline.

Kaplan–Meier survival curve analysis (Figure 3) graphically illustrated significant differences in the risk of incident IFG among the four groups stratified by CHG index quartiles. The IFG incidence progressively increased from the Q1 to the Q4 group, with the Q4 group exhibiting the highest incidence. The result of the log-rank test was statistically significant (*p* < 0.0001), which is consistent with the findings from the stacked bar chart of baseline CHG index groups (Figure 4). Both analyses provide evidence that a higher CHG index is associated with an increased risk of developing IFG.

Regarding model performance, the C-statistic for the fully adjusted model incorporating the baseline CHG index was 0.6075 (95% CI: 0.6022**–**0.6128). Furthermore, the calibration plot (Supplementary Figure 2) demonstrated moderate agreement between the predicted and observed probabilities of incident IFG. While the calibration curve aligned reasonably well with the ideal reference line within the intermediate risk ranges (predicted probabilities between 40% and 75%), noticeable deviations were observed at the extremes, particularly in the lower predicted probability ranges where the model tended to underestimate the actual risk. Overall, the model exhibited acceptable calibration for risk assessment.

### Nonlinear relationship and threshold effect analysis

**Figure 5:**
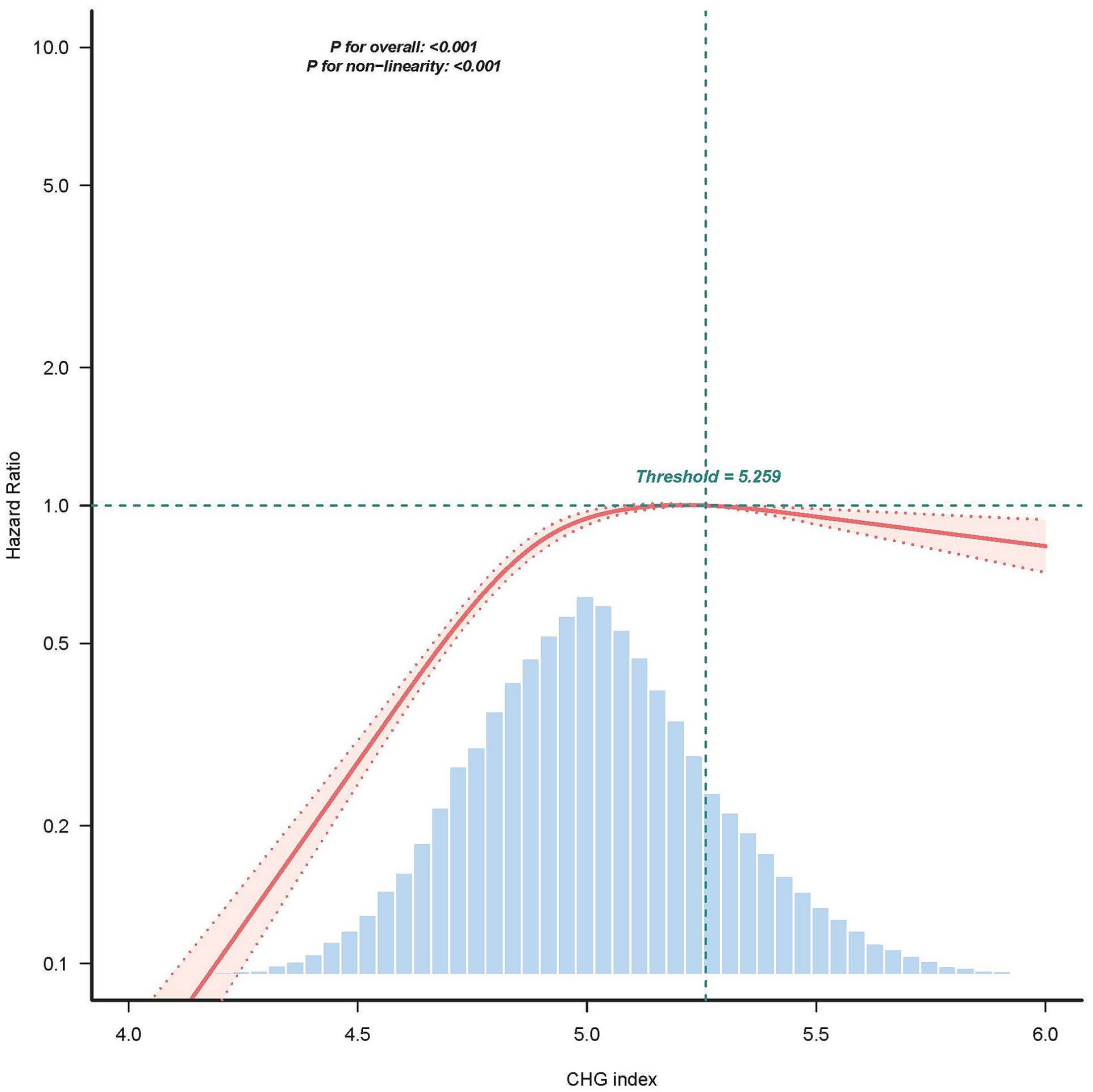
The nonlinear relationship between CHG index and risk of incident IFG. We adjusted age, gender, height, body weight, SBP, DBP, ALT, AST, TG, LDL-C, BUN, Scr, smoking status, and family history of DM at baseline.

Analysis using an RCS model revealed a significant nonlinear association between the baseline CHG index and the risk of incident IFG (*P* for nonlinearity < 0.001). As depicted in Figure 5, the relationship between the CHG index and IFG risk was not a simple linear one but exhibited a distinct threshold effect. With an inflection point at a CHG index of 5.259, the risk association demonstrated different patterns. When the CHG index was below this inflection point, it showed a significant positive correlation with the risk of incident IFG (HR = 4.552, 95% CI: 4.009–5.170, *p* < 0.001). However, when the CHG index reached or exceeded 5.259, the strength of this association was significantly attenuated and no longer statistically significant (HR = 1.329, 95% CI: 0.991–1.782, *p* = 0.0575) (Table 5).

**Table 5:** Analysis of the critical inflection point governing the CHG index and the risk of IFG.

CHG index	HR (95% CI)	*p* value
< 5.259	4.552 (4.009, 5.17)	< 0.001
≥ 5.259	1.329 (0.991, 1.782)	0.0575
*P* for Likelihood Ratio test		< 0.001

We adjusted age, gender, height, body weight, SBP, DBP, ALT, AST, TG, LDL-C, BUN, Scr, smoking status, and family history of DM at baseline.

### Subgroup analysis

**Figure 6:**
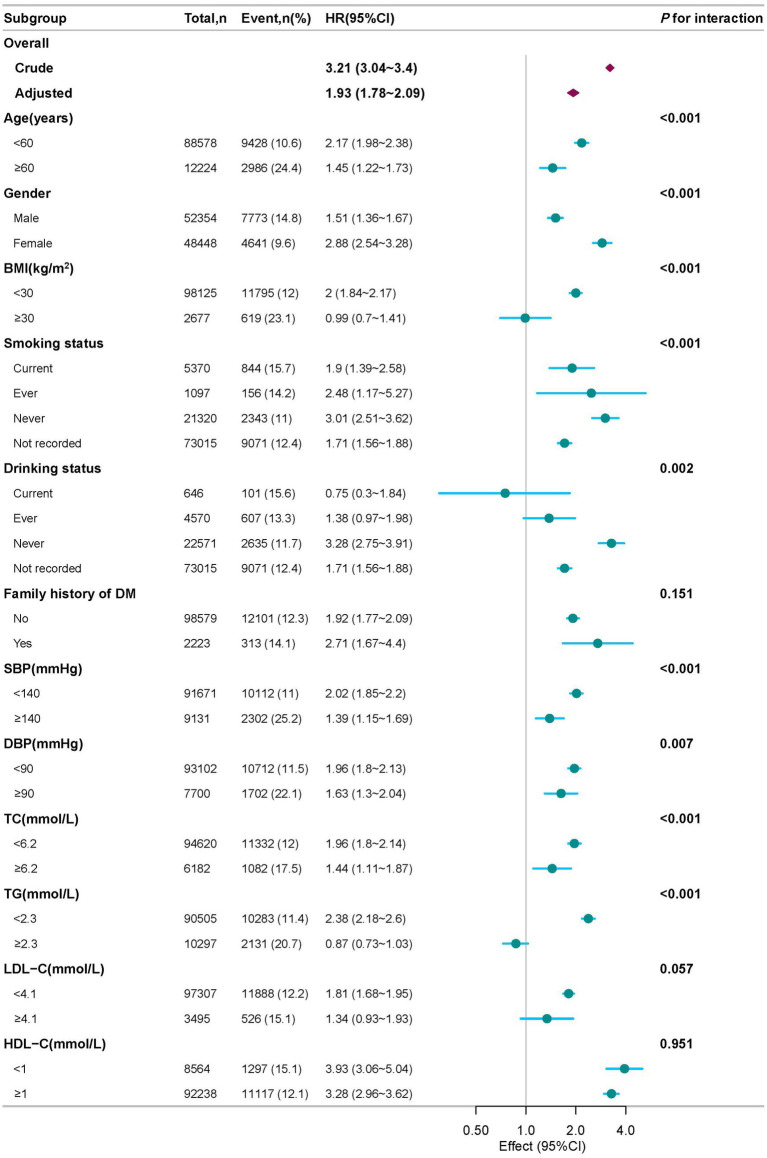
Forest plot of subgroup analysis of the association between CHG index and risk of incident IFG.

The results of the subgroup analysis are presented in the forest plot (Figure 6). Across all subgroups stratified by age, gender, BMI, family history of DM, smoking and drinking status, SBP, DBP, TG, TC, HDL-C, and LDL-C, the CHG index consistently showed a positive association with the risk of incident IFG, with the vast majority of HRs exceeding 1. This indicates that the positive correlation between the CHG index and IFG risk remains consistent across populations with diverse characteristics.

### Sensitivity analysis

To ensure the robustness of the study findings, sensitivity analyses were performed. First, analyses were conducted using the original dataset containing all participants (Supplementary Tables 2, 3). The results from both cox and logistic regression models were largely consistent with the primary findings, indicating that a higher CHG index was significantly associated with an increased risk of incident IFG (in model II of the cox regression, HR = 3.74, 95% CI: 2.88–4.85, *p* < 0.001; in model II of the logistic regression, OR = 5.05, 95% CI: 3.71–6.87, *p* < 0.001). Second, after excluding participants with incomplete covariate data (*n* = 10,540), the analysis results (Supplementary Tables 4, 5) showed that the association between the CHG index and IFG risk remained significant, with effect sizes slightly strengthened compared to the main analysis (in model II of the cox regression, HR = 3.74, 95% CI: 2.88–4.85; in model II of the logistic regression, OR = 5.05, 95% CI: 3.71–6.87). The consistency of these results further supports the reliability of the primary findings.

Within the original dataset containing all participants, an RCS model was also employed to evaluate the dose–response relationship between the CHG index and the risk of incident IFG (Supplementary Figure 3). After adjusting for age, gender, height, body weight, SBP, DBP, ALT, AST, TG, LDL-C, BUN, Scr, smoking status, and family history of DM, a significant nonlinear association was observed between the CHG index and the risk of incident IFG (*P* for nonlinearity < 0.001). A significant nonlinear association was similarly observed in the dataset where participants with incomplete covariate data were excluded (Supplementary Figure 4, *P* for nonlinearity < 0.001). The consistency of these analytical results further reinforces the reliability of the main findings. Furthermore, we assessed the potential impact of unmeasured confounding factors by calculating the E-value. The results indicated that if there were an unmeasured confounding factor, its relative risk association with both the CHG index and incident IFG would need to reach 3.27 to fully explain away the observed association (HR = 1.93) in the fully adjusted model.

## Discussion

Based on a secondary analysis of a large-scale Chinese population cohort, this study first systematically evaluated the association between the baseline CHG index and the risk of incident IFG among adults with normal fasting glucose. Among the 100,802 Chinese adults with normal baseline fasting glucose included, a total of 12,414 participants developed incident IFG during follow-up. Multivariable Cox proportional hazards regression analysis demonstrated that a higher baseline CHG index was an independent risk factor for incident IFG. In the fully adjusted model, each one-unit increase in the CHG index was associated with an HR of 1.93 for incident IFG. Analysis by CHG index quartiles further revealed a significant dose–response relationship. Notably, RCS model analysis identified a significant nonlinear association between the baseline CHG index and the risk of incident IFG, with a distinct threshold effect: with an inflection point at a CHG index of 5.259, the risk increased sharply below this value, while the association strength significantly attenuated and became statistically non-significant at or above this threshold. This positive association remained consistent across subgroups stratified by age, gender, BMI, blood pressure, smoking status, drinking status, family history of DM, and different lipid levels (TG, TC, HDL-C, and LDL-C), and the results were robust as validated by sensitivity analyses. The findings of this study indicate that the baseline CHG index is a strong predictor of incident IFG in the Chinese adult population, and its association exhibits a nonlinear threshold characteristic, providing a more nuanced perspective for IFG risk assessment.

This study found that the CHG index is an independent predictor of incident IFG in Chinese adults with normal baseline glucose, a conclusion consistent with the recent trend establishing the CHG index as an effective marker for IR and metabolic dysregulation. As a novel composite metric integrating TC, FPG, and HDL-C, the CHG index simultaneously captures core information on atherogenic dyslipidemia (high TC, low HDL-C) and dysglycemia (high FPG). Theoretically, it provides a more comprehensive assessment of an individual’s metabolic dysregulation than a single parameter (19, 23). Multiple studies support the predictive value of the CHG index for DM. Cross-sectional research has shown a significant association between the CHG index and the risk of prevalent DM, with diagnostic performance comparable to or even superior to the classic TyG index (19, 22, 23). Regarding diabetic complications, an elevated CHG index is independently associated with an increased risk of DR and DN (24, 28). Several studies have confirmed that elevated baseline or cumulative CHG indices can predict future risks of CVD, stroke, and all-cause mortality (20, 25, 29, 30). The present study advances the predictive window of the CHG index to the IFG stage, clarifying its ability to predict the entire continuum of glucose metabolism deterioration. Given that IFG precedes DM, our results indicate that the CHG index also serves as a sensitive early warning marker at this earlier stage of glucose homeostasis disruption. Compared to traditional predictors of IFG risk (such as age, obesity, hypertension, and family history) and single lipid parameters, the strength of the CHG index lies in its integrative nature. In this study, the association between the CHG index and IFG remained significant in the fully adjusted model, even after controlling for various conventional risk factors, including body weight, SBP, smoking, and lipid levels. This suggests that the CHG index may provide additional risk information beyond traditional factors, stemming from the interplay between glucose and lipid metabolism. This aligns with findings from other studies demonstrating the unique value of the CHG index in predicting MetS and its associated mortality risk (21). The nonlinear association between the CHG index and IFG risk observed in this study is a noteworthy feature. Existing research on the pattern of association between the CHG index and disease outcomes is not entirely consistent, reporting linear (20, 25), U-shaped (21, 31), or J-shaped relationships (32). These discrepancies may stem from differences in study populations, underlying disease status, and outcome measures. In this study, the inflection point for the CHG index in relation to IFG risk was 5.259. The risk increased sharply when the CHG index was below this threshold, while the risk increase plateaued beyond it. This finding suggests the potential existence of a critical point of metabolic disturbance in the development of IFG, which may have implications for clinical risk stratification. However, the underlying biological mechanisms require further exploration.

The association between the CHG index and IFG risk is biologically plausible, primarily driven by interconnected metabolic disturbances. IR likely serves as the central nexus, where elevated TC and reduced HDL-C are hallmarks of dyslipidemia closely tied to systemic IR and elevated FPG (33). Furthermore, glucolipotoxicity and chronic low-grade inflammation may synergistically exacerbate IR and impair pancreatic *β*-cell function (34–36). Additionally, low HDL-C levels may indicate a loss of its protective anti-inflammatory and antioxidant properties, rendering tissues more susceptible to metabolic damage (37, 38). Regarding the observed nonlinear threshold effect, we hypothesize it may reflect a critical point of metabolic decompensation, beyond which compensatory mechanisms (such as increased insulin secretion) are progressively overwhelmed. However, as the present study is observational, these underlying biological pathways and specific molecular mechanisms require further elucidation through future experimental and mechanistic research. In summary, the CHG index, by integrating multiple dimensions reflecting IR (high FPG), an atherogenic lipid profile (high TC, low HDL-C), and potential inflammation/dysfunction, emerges as a composite marker capable of capturing early disruptions in the glucose-lipid metabolic network, thereby effectively predicting the development of IFG.

The findings of this study provide a novel and simple tool for the early identification and prevention of IFG. The CHG index can be calculated solely based on routine lipid and fasting glucose tests, requiring no additional cost or invasive procedures. This grants it high operability and scalability for clinical practice and population screening. For individuals with normal baseline glucose, particularly those identified with dyslipidemia during health checks, calculating their CHG index can serve as an effective supplementary measure for assessing the future risk of worsening glucose metabolism (39). At the clinical level, the identified risk threshold of 5.259 holds significant translational value for risk stratification and guiding personalized interventions. Practitioners can utilize this specific cutoff as a metabolic “alert line” for individuals with currently normal fasting glucose. Specifically, the application is two-fold: First, for individuals with a CHG index below 5.259, practitioners should recognize this as a “dynamic deterioration zone” where even slight increases in the index correspond to a sharply elevated risk of incident IFG. In this phase, early prophylactic measures—such as intensive dietary modifications and prescribed physical activity—yield the highest preventive benefit. Second, when an individual’s CHG index approaches or exceeds the 5.259 threshold, practitioners should consider their metabolic compensatory mechanisms to be highly strained. For these high-risk patients, routine annual health checks are insufficient. Practitioners should implement more aggressive management strategies, including significantly shortened intervals for follow-up monitoring, the addition of oral glucose tolerance tests (OGTT) or HbA1c measurements to rule out hidden dysglycemia, and earlier referral to metabolic specialists if lipid and glucose profiles do not improve with lifestyle changes. This targeted approach aids in achieving precision primary prevention by moving the intervention point forward based on an objective, easily calculable parameter. Furthermore, the integrated glucolipid metabolic information captured by the CHG index may better reflect the overall burden of metabolic dysregulation than single parameters, assisting clinicians in conducting more comprehensive risk assessments (39). At the public health level, the CHG index, validated in a large-scale population cohort, can serve as a reference for developing community-based screening strategies for prediabetes (20). In resource-limited settings, incorporating CHG index calculation into routine health examinations for middle-aged and older adults could be considered to more efficiently identify target populations requiring focused intervention, thereby optimizing the allocation of public health resources. These findings further reinforce the public health concept that lipid management and glycemic control are inseparable, supporting the improvement of lipid profiles as an integral component of a comprehensive DM prevention strategy. It is important to note that, although the CHG index demonstrates good predictive performance, its value as an independent decision-making tool in clinical settings requires further validation in prospective studies. Future research should also assess whether interventions based on this index can ultimately reduce the incidence of IFG and DM. Subsequent studies could explore combining the CHG index with other risk factors (e.g., genetic markers, imaging indicators) to construct more precise risk prediction models.

This study has several notable strengths. Firstly, it is a secondary analysis based on a large-scale, prospectively designed Chinese population cohort with an adequate sample size (n = 100,802), ensuring statistical power and enhancing the generalizability of the findings. Secondly, the study strictly enrolled participants with normal baseline fasting glucose, establishing a clear temporal sequence between the exposure (CHG index) and the outcome (incident IFG), which aligns with the fundamental requirements for causal inference. Thirdly, we employed multivariable Cox regression, restricted cubic spline models, and extensive subgroup and sensitivity analyses to comprehensively assess the strength, pattern, and robustness of the association. Finally, the CHG index, as an objective laboratory-derived measure, avoids recall bias and is simple to calculate, facilitating clinical translation. However, as an observational study, it also has inherent limitations. The primary limitation is the possibility of residual confounding. Although we adjusted for a wide range of demographic, lifestyle, and clinical covariates, the influence of unmeasured or imprecisely measured confounders (such as detailed dietary patterns, intensity of physical activity, genetic background, or subtle socioeconomic differences) on the association cannot be entirely ruled out. However, based on our E-value sensitivity analysis, an unmeasured confounder would need a relative risk of at least 3.27 with both the CHG index and IFG to completely explain away the observed association, suggesting our primary findings are relatively robust to unmeasured confounding. Secondly, the conclusions are based on the CHG index measured at a single time point (baseline). The study did not examine the dynamic changes in the CHG index over time or its association with IFG risk, which may limit the precision of predicting an individual’s long-term risk trajectory. Thirdly, while the outcome was defined as a single FPG measurement ≥ 5.6 mmol/L and < 7.0 mmol/L, consistent with commonly used diagnostic criteria, the lack of a requirement for repeated confirmatory testing may introduce some degree of misclassification bias. Furthermore, as a retrospective cohort study, data on some potentially important covariates (such as insulin levels, glycated hemoglobin, and detailed medication history) may be missing. Fifthly, to isolate the specific transition to early prediabetes, we excluded participants who developed overt diabetes during follow-up; while consistent with previous studies using this dataset, this methodological choice may introduce a degree of selection bias or overlook the competing risk of rapid diabetes progression. Sixthly, we did not perform head-to-head comparative analyses evaluating the predictive value of the CHG index against other established surrogate indicators of IR, such as the TyG index, the atherogenic index of plasma (AIP), and the metabolic score for insulin resistance (METS-IR). Because determining the optimal predictive biomarker requires complex comparative methodologies, this evaluation fell outside the primary scope of the current investigation but represents a critical direction for future evaluation. Finally, the observational design inherently restricts our ability to establish definitive causality. While the temporal sequence is maintained by strictly excluding individuals with baseline IFG, we cannot completely exclude the possibility of reverse causation or parallel progression driven by shared upstream mechanisms. Validating a true causal relationship requires future investigations utilizing Mendelian randomization to leverage genetic instruments, alongside well-designed prospective randomized controlled trials (RCTs).

Building on the findings and limitations of this study, future research can be deepened across multiple dimensions. First, prospective intervention studies are needed to test causal hypotheses. For example, designing randomized controlled trials to evaluate whether lowering the CHG index through lifestyle or pharmacological interventions can effectively delay or prevent the progression from normal fasting glucose to IFG or DM. Second, longitudinal data should be utilized to investigate the dynamic trajectory of the CHG index. Analyzing its patterns of change over time and the association between its rate of change and IFG risk could provide more precise risk prediction information than a single measurement. Third, mechanistic exploration requires further deepening. Future studies could integrate multi-omics technologies (e.g., metabolomics, proteomics) and functional experiments to elucidate how the specific metabolic disturbance network represented by the CHG index concretely leads to IR and *β*-cell dysfunction. Fourth, the global generalizability of our findings warrants further discussion and investigation. The CHG index formula was originally developed and validated in a Middle Eastern population (19). Our study provides robust longitudinal evidence extending its predictive utility to a large Chinese cohort. This trans-ethnic consistency suggests that the core biological relationship captured by the CHG index—the interplay between dyslipidemia and dysglycemia—likely applies to populations outside of China. However, due to inherent differences in genetic backgrounds, dietary patterns, and baseline lipid profiles across diverse ethnic groups, the specific optimal threshold identified in our study may not be universally applicable. Therefore, future studies require external validation in other ethnicities and geographical regions (e.g., Western populations) to determine whether ethnicity-specific cutoffs are necessary for precise clinical application. Fifth, future studies should conduct comprehensive comparative analyses to rigorously evaluate the predictive performance of the CHG index against other surrogate indicators like the TyG index, AIP, and METS-IR. Such comparisons will be crucial for identifying the most robust and clinically optimal biomarker for early IFG risk stratification. Finally, promoting translational clinical research is crucial. This includes developing and validating practical tools that integrate the CHG index into existing DM risk scores, as well as evaluating the health economic benefits of risk stratification and early intervention based on this index, thereby providing empirical evidence for public health decision-making.

## Conclusion

This study, for the first time in a large-scale Chinese adult population with normal fasting glucose, confirms that the baseline CHG index is an independent and strong predictor of incident IFG, and this association exhibits a significant nonlinear threshold characteristic. This finding expands the scope of application for the CHG index in the early warning of glucose metabolism abnormalities. The results provide important epidemiological evidence for utilizing this simple and economical composite index to early identify individuals at high risk for IFG and to implement targeted interventions at both clinical and public health levels. Future prospective intervention studies and in-depth mechanistic investigations are needed to further validate its causal role and promote clinical translation.

## Data Availability

Publicly available datasets were analyzed in this study. This data can be found here: the dataset presented in this study is available in the online repository (https://doi.org/10.5061/dryad.ft8750v).

## Glossary

CHG: Cholesterol, high-density lipoprotein, and glucose (index)
DM: Diabetes mellitus
IDF: International Diabetes Federation
IFG: Impaired fasting glucose
FPG: Fasting plasma glucose
CVD: Cardiovascular disease
DR: Diabetic retinopathy
DN: Diabetic nephropathy
IR: Insulin resistance
MetS: Metabolic syndrome
TyG: Triglyceride-glucose (index)
TG: Triglycerides
TC: Total cholesterol
HDL-C: High-density lipoprotein cholesterol
ADA: American Diabetes Association
BP: Blood pressure
LDL-C: Low-density lipoprotein cholesterol
ALT: Alanine aminotransferase
AST: Aspartate aminotransferase
BUN: Blood urea nitrogen
Scr: Serum creatinine
BMI: Body mass index
MICE: Multivariate Imputation by Chained Equations
M (IQR): Median (interquartile range)
SD: Standard deviation
HR: Hazard ratio
CI: Confidence interval
OR: Odds ratio
RCS: Restricted cubic splines
STROBE: Strengthening the Reporting of Observational Studies in Epidemiology
SBP: Systolic blood pressure
DBP: Diastolic blood pressure
PI3K/Akt: Phosphoinositide 3-kinase/Protein kinase B
TNF-α: Tumor necrosis factor-α
IL-6: Interleukin-6
