# Supplementary material for: Cholesterol, high-density lipoprotein, and glucose (CHG) index and incident impaired fasting glucose in Chinese adults: a retrospective longitudinal cohort study
Source: Front Nutr. 2026 Jun 2;13:1814747. doi: 10.3389/fnut.2026.1814747 (PMC13270551; doi:10.3389/fnut.2026.1814747)

**Supplementary Table 1 Description of missing data.**

| **Variables** | **Non-missing** | **Missing** | **Miss percentage (%)** |
| --- | --- | --- | --- |
| Age | 100802 | 0 | 0 |
| Gender | 100802 | 0 | 0 |
| Height | 100802 | 0 | 0 |
| Body weight | 100802 | 0 | 0 |
| BMI | 100802 | 0 | 0 |
| Baseline FPG | 100802 | 0 | 0 |
| ALT | 100430 | 372 | 0.37 |
| AST | 42296 | 58506 | 58.04 |
| BUN | 98494 | 2308 | 2.29 |
| Scr | 99585 | 1217 | 1.21 |
| SBP | 100789 | 13 | 0.01 |
| DBP | 100789 | 13 | 0.01 |
| HDL-C | 100802 | 0 | 0 |
| LDL-C | 100640 | 162 | 0.16 |
| TC | 100802 | 0 | 0 |
| TG | 100800 | 2 | 0.002 |
| Family histroy of DM | 100802 | 0 | 0 |
| Drinking status | 27787 | 73015 | 72.43 |
| Smoking status | 27787 | 73015 | 72.43 |

**Supplementary Table 2 Association between CHG index and the risk of incident IFG, assessed using​ Cox proportional hazards regression models in​ the original dataset**

| Variables | Crude model | | Model Ⅰ | | Model Ⅱ | |
| --- | --- | --- | --- | --- | --- | --- |
|  | HR (95% CI) | *P*-Value | HR (95% CI) | *P*-Value | HR (95% CI) | *P*-Value |
| CHG index | 3.21 (3.04, 3.4) | < 0.001 | 1.83 (1.71, 1.95) | < 0.001 | 3.74 (2.88, 4.85) | < 0.001 |
| (CHG index quartiles) |  |  |  |  |  |  |
| Q1 | 1.00 (Reference) |  | 1.00 (Reference) |  | 1.00 (Reference) |  |
| Q2 | 2.05 (1.92, 2.19) | < 0.001 | 2.05 (1.92, 2.19) | < 0.001 | 2.01 (1.6, 2.53) | < 0.001 |
| Q3 | 2.9 (2.73, 3.08) | < 0.001 | 2.9 (2.73, 3.08) | < 0.001 | 3.07 (2.46, 3.84) | < 0.001 |
| Q4 | 3.17 (2.99, 3.37) | < 0.001 | 3.17 (2.99, 3.37) | < 0.001 | 3.5 (2.77, 4.42) | < 0.001 |
| *P* for trend |  | < 0.001 |  | < 0.001 |  | < 0.001 |

Crude model: we did not adjust other covariates.
Model I: adjusted for age, gender, height, body weight, and family history of DM at baseline.

Model II: further adjusted for SBP, DBP, ALT, AST, TG, LDL-C, BUN, Scr, smoking status, and drinking status at baseline.

**Supplementary Table 3 Association between CHG index and incident IFG, assessed using​ logistic​ regression models in​ the original dataset.**

| Variables | Crude model | | Model Ⅰ | | Model Ⅱ | |
| --- | --- | --- | --- | --- | --- | --- |
|  | OR (95% CI) | *P*-Value | OR (95% CI) | *P*-Value | OR (95% CI) | *P*-Value |
| CHG index | 5.26 (4.92, 5.62) | < 0.001 | 2.6 (2.41, 2.8) | < 0.001 | 5.05 (3.71, 6.87) | < 0.001 |
| (CHG index quartiles) |  |  |  |  |  |  |
| Q1 | 1.00 (Reference) |  | 1.00 (Reference) |  | 1.00 (Reference) |  |
| Q2 | 1.82 (1.7, 1.95) | < 0.001 | 1.47 (1.37, 1.58) | < 0.001 | 1.48 (1.16, 1.88) | 0.001 |
| Q3 | 2.88 (2.7, 3.07) | < 0.001 | 1.96 (1.83, 2.1) | < 0.001 | 2.2 (1.74, 2.78) | < 0.001 |
| Q4 | 3.92 (3.68, 4.17) | < 0.001 | 2.22 (2.07, 2.37) | < 0.001 | 3.19 (2.49, 4.1) | < 0.001 |
| *P* for trend |  | < 0.001 |  | < 0.001 |  | < 0.001 |

Crude model: we did not adjust other covariates.
Model I: adjusted for age, gender, height, body weight, and family history of DM at baseline.

Model II: further adjusted for SBP, DBP, ALT, AST, TG, LDL-C, BUN, Scr, smoking status, and drinking status at baseline.

**Supplementary Table 4 Association between CHG index and the risk of incident IFG, assessed using​ Cox proportional hazards regression models after excluding participants with incomplete covariate data.**

| Variables | Crude model | | Model Ⅰ | | Model Ⅱ | |
| --- | --- | --- | --- | --- | --- | --- |
|  | HR (95% CI) | *P*-Value | HR (95% CI) | *P*-Value | HR (95% CI) | *P*-Value |
| CHG index | 4.67 (3.92, 5.57) | < 0.001 | 2.83 (2.32, 3.45) | < 0.001 | 3.74 (2.88, 4.85) | < 0.001 |
| (CHG index quartiles) |  |  |  |  |  |  |
| Q1 | 1.00 (Reference) |  | 1.00 (Reference) |  | 1.00 (Reference) |  |
| Q2 | 2.31 (1.88, 2.85) | < 0.001 | 2.03 (1.65, 2.51) | < 0.001 | 2.09 (1.69, 2.59) | < 0.001 |
| Q3 | 3.7 (3.04, 4.5) | < 0.001 | 2.82 (2.31, 3.45) | < 0.001 | 3.01 (2.44, 3.71) | < 0.001 |
| Q4 | 4.31 (3.57, 5.2) | < 0.001 | 2.88 (2.36, 3.51) | < 0.001 | 3.19 (2.55, 3.98) | < 0.001 |
| *P* for trend |  | < 0.001 |  | < 0.001 |  | < 0.001 |

Crude model: we did not adjust other covariates.
Model I: adjusted for age, gender, height, body weight, and family history of DM at baseline.

Model II: further adjusted for SBP, DBP, ALT, AST, TG, LDL-C, BUN, Scr, smoking status, and drinking status at baseline.

**Supplementary Table 5 Association between CHG index and incident IFG, assessed using​ logistic​ regression models after excluding participants with incomplete covariate data.**

| Variables | Crude model | | Model Ⅰ | | Model Ⅱ | |
| --- | --- | --- | --- | --- | --- | --- |
|  | OR (95% CI) | *P*-Value | OR (95% CI) | *P*-Value | OR (95% CI) | *P*-Value |
| CHG index | 6.95 (5.59, 8.64) | < 0.001 | 3.86 (3.03, 4.93) | < 0.001 | 5.05 (3.71, 6.87) | < 0.001 |
| (CHG index quartiles) |  |  |  |  |  |  |
| Q1 | 1.00 (Reference) |  | 1.00 (Reference) |  | 1.00 (Reference) |  |
| Q2 | 1.81 (1.46, 2.25) | < 0.001 | 1.55 (1.25, 1.93) | < 0.001 | 1.63 (1.3, 2.03) | < 0.001 |
| Q3 | 2.97 (2.42, 3.63) | < 0.001 | 2.15 (1.74, 2.65) | < 0.001 | 2.31 (1.85, 2.88) | < 0.001 |
| Q4 | 4.45 (3.66, 5.41) | < 0.001 | 2.82 (2.28, 3.48) | < 0.001 | 3.06 (2.42, 3.88) | < 0.001 |
| *P* for trend |  | < 0.001 |  | < 0.001 |  | < 0.001 |

Crude model: we did not adjust other covariates.
Model I: adjusted for age, gender, height, body weight, and family history of DM at baseline.

Model II: further adjusted for SBP, DBP, ALT, AST, TG, LDL-C, BUN, Scr, smoking status, and drinking status at baseline.

**Supplementary Figure 1. Schoenfeld residual plot for assessing the proportional hazards assumption of the Cox model.**


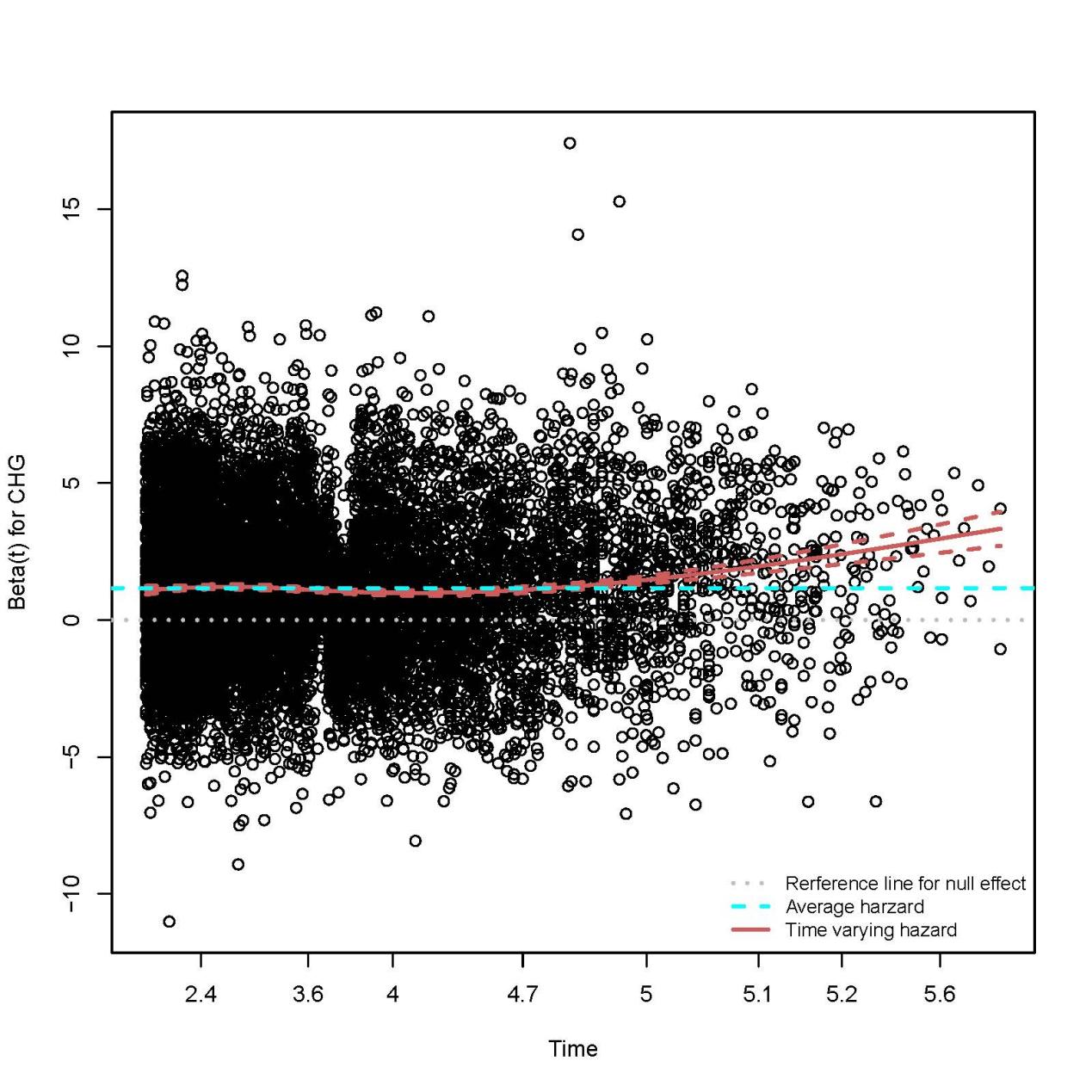


**Supplementary Figure 2. Calibration plot of the fully adjusted model for predicting incident IFG.**


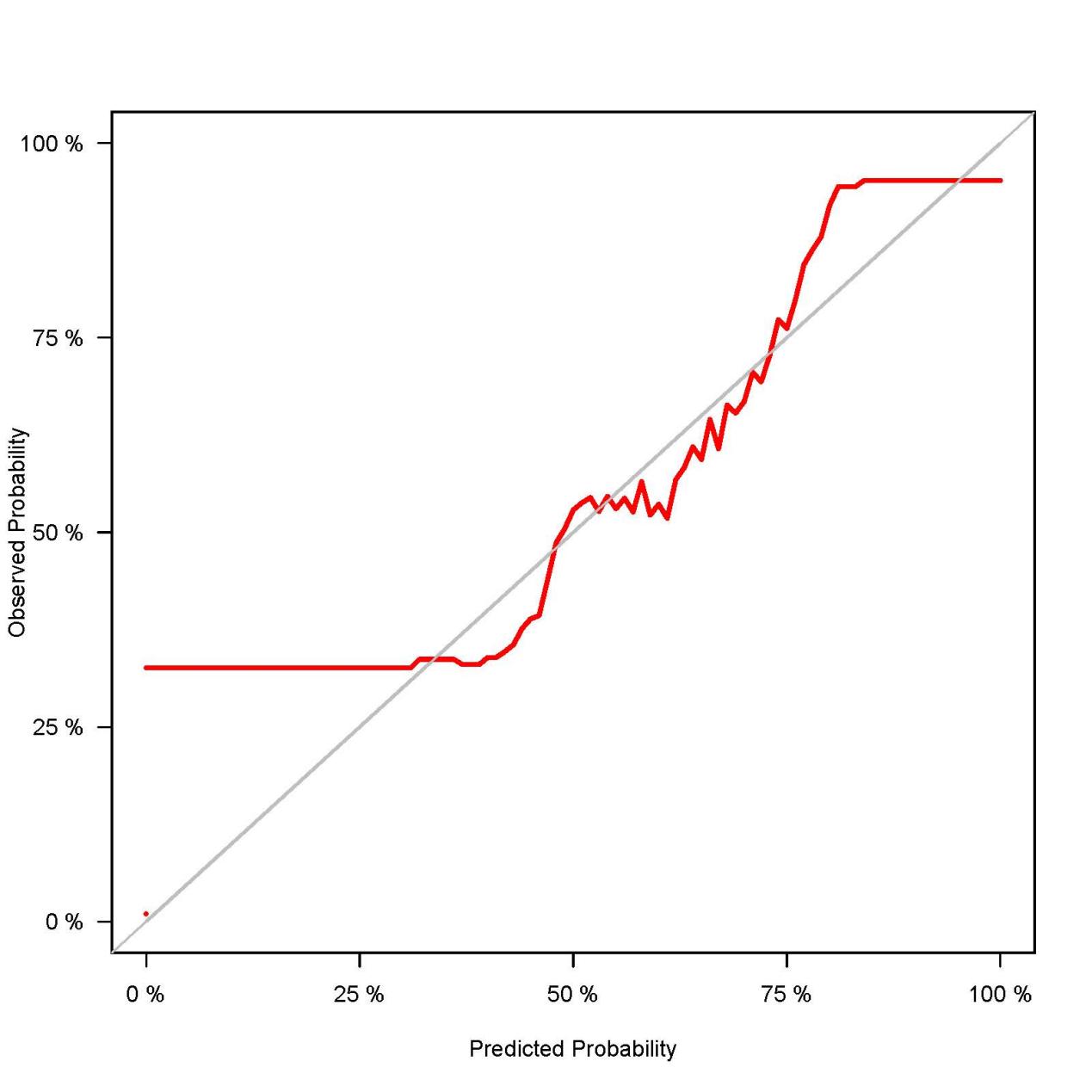


**Supplementary Figure 3. The non-linear relationship between CHG index and risk of incident IFG using the original dataset.** We adjusted age, gender, height, body weight, SBP, DBP, ALT, AST, TG, LDL-C, BUN, Scr, smoking status, and family history of diabetes at baseline.


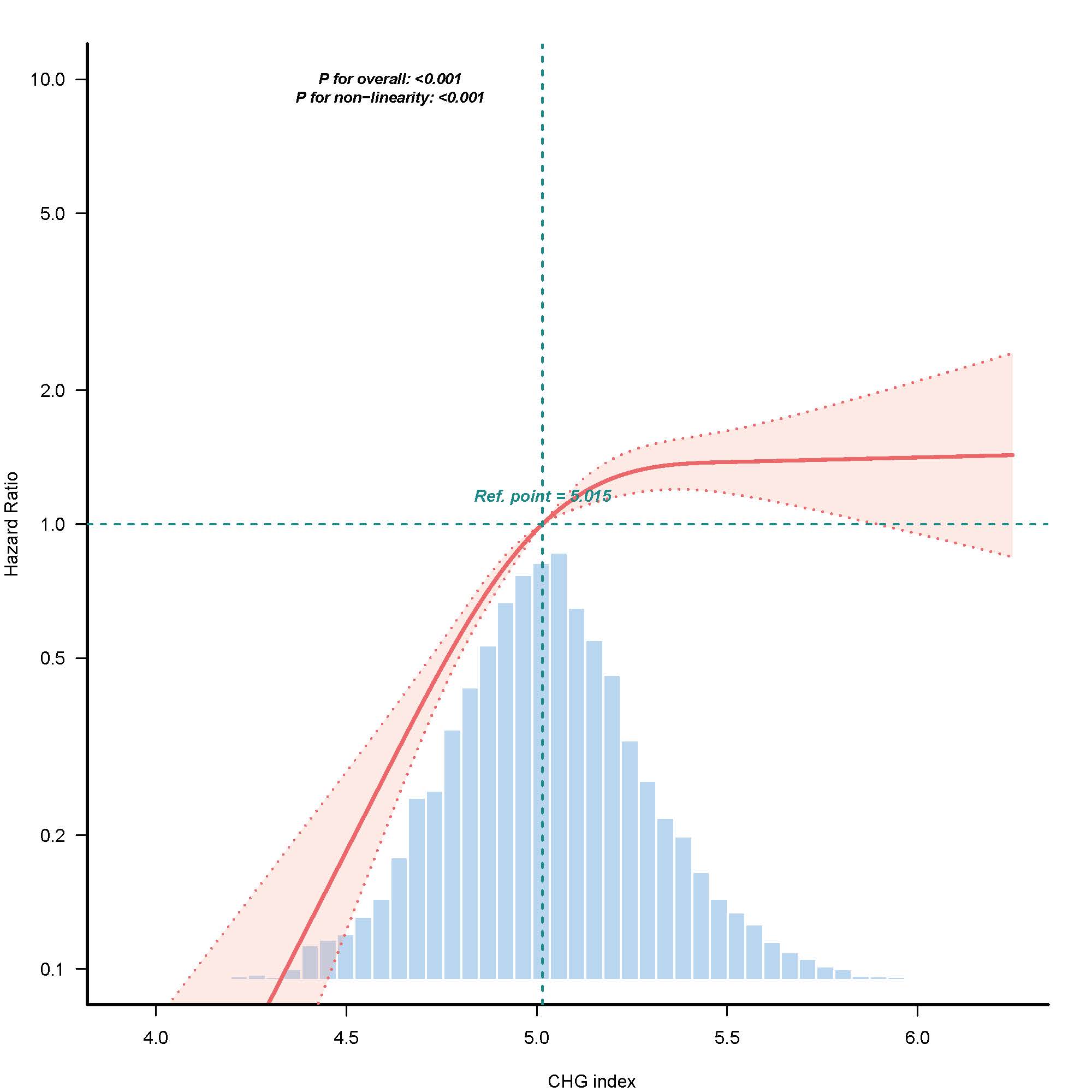


.

**Supplementary Figure 4. The non-linear relationship between CHG index and risk of incident IFG using the excluding participants with incomplete covariate data.** We adjusted age, gender, height, body weight, SBP, DBP, ALT, AST, TG, LDL-C, BUN, Scr, smoking status, and family history of diabetes at baseline.


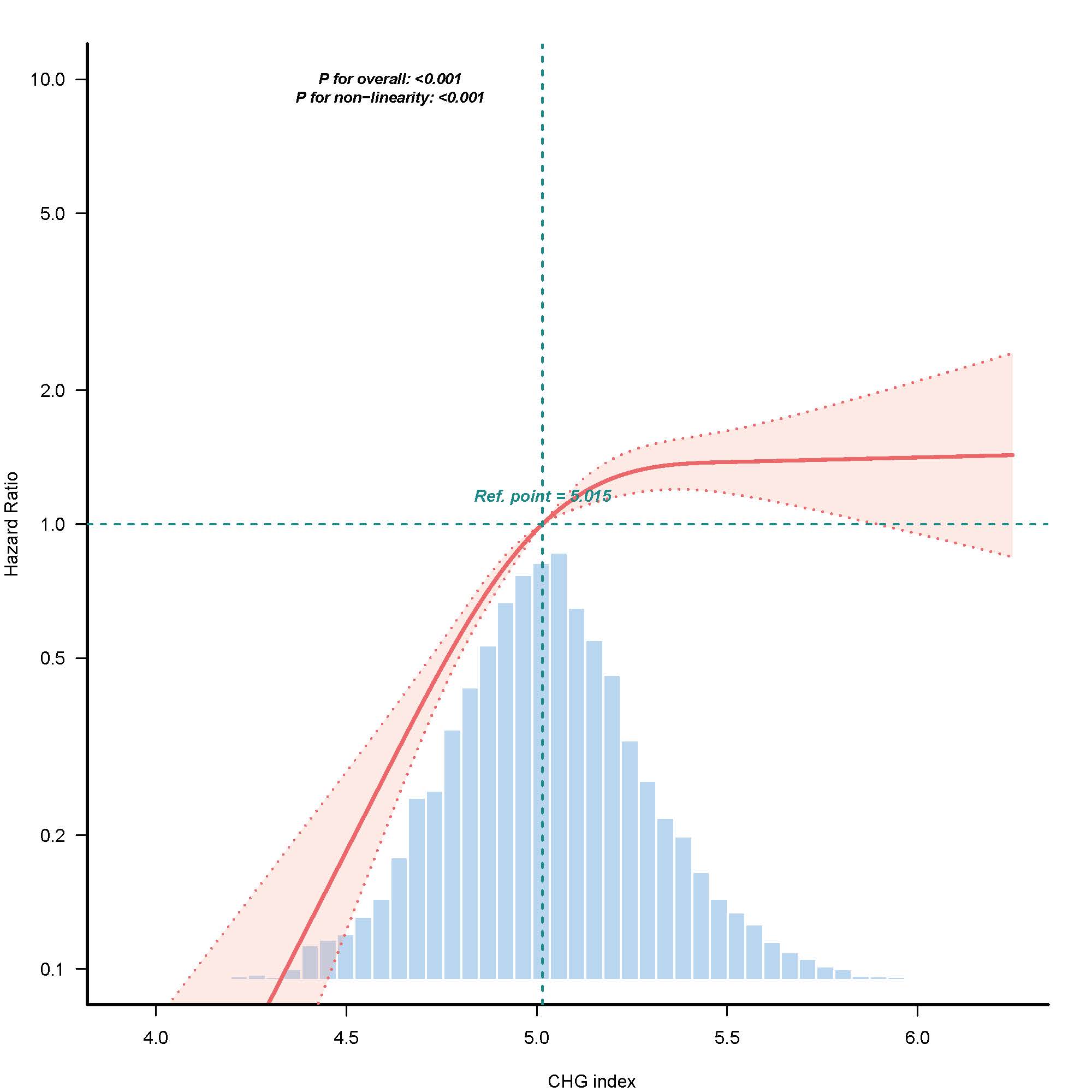

Supplement: Supplementary file 1 [file Table_1.docx]
